# Acupuncture for COVID-19 patient after ventilator weaning: A protocol for systematic review and meta-analysis

**DOI:** 10.1097/MD.0000000000023602

**Published:** 2020-12-11

**Authors:** Wenxin Chi, Ying Chen, Lina Wang, Ziyu Luo, Yu Zhang, Xiangyu Zhu

**Affiliations:** a School of Acupuncture, Moxibustion and Tuina, Beijing University of Chinese Medicine; b Dongzhimen Hospital, Beijing University of Chinese Medicine; c Laboratory of Statistics and Measurement, Beijing Sport University, Beijing, China.

**Keywords:** acupuncture, COVID-19, meta-analysis, systematic review, ventilator weaning

## Abstract

**Background:**

COVID-19 has spread globally since its outbreak in late 2019. It mainly attacks people's respiratory system. Many patients with severe COVID-19 require a ventilator to support breathing, and their lung function is often impaired to varying degrees after ventilator weaning. Acupuncture has been reported to improve respiratory function, but there is no evidence that it can improve respiratory function in ventilator users with COVID-19 after they are removed from the machine. The protocol of the systematic review and meta-analysis will clarify safety and effectiveness of acupuncture on respiratory rehabilitation after weaning from the ventilator during the treatment of COVID-19.

**Methods:**

We will search PubMed, EMBASE, MEDLINE, the Cochrane Library, Chinese National Knowledge Infrastructure, Chinese Biomedical Literature Database, Chinese Science and Technology Periodical Database, Wanfang Database, Clinical Trials and Chinese Clinical Trial Registry. Relevant English language and Chinese language literature will be included. A combination of subject words and free text words will be applied in the searches. The complete process will include study selection, data extraction, risk of bias assessment, and meta-analyses. We will use subgroup analysis and sensitivity analysis to explore the sources of heterogeneity if there is heterogeneity. We will use funnel charts to assess the risk of bias. Endnote X9.3 will be used to manage data screening. The statistical analysis will be completed by RevMan5.2 or Stata/SE 15.1 software.

**Results:**

This study will assess safety and effectiveness of acupuncture for rehabilitation on respiratory function after weaning from the ventilator during the treatment of COVID-19.

**Conclusions:**

The conclusion of this study will give evidence to prove safety and effectiveness of acupuncture for rehabilitation on respiratory after weaning from the ventilator during the treatment of COVID-19.

**Registration:**

PROSPERO CRD42020206889

## 1 Introduction

A novel coronavirus (COVID-19) was found to cause a highly contagious disease characterized by pneumonia in late 2019. The disease (COVID-19) quickly spread around the globe, escalating to a global pandemic.^[1–2]^ At the time of writing (December 2020), more than 34 million cases had been reported and more than 1,030,000 patients had succumbed to the disease worldwide. It is a highly contagious respiratory disease characterized by low fever, cough, airway obstruction, dyspnea, chest pain, and so on.^[3]^ Also, there is growing evidence that COVID-19 patients have a severe pulmonary embolism burden.^[4]^ Several typical imaging features are frequently observed in COVID-19 pneumonia, including predominant ground-glass opacity (65%), consolidations (50%), smooth or irregular interlobular septal thickening (35%), air bronchogram (47%), and thickening of the adjacent pleura (32%), with predominantly peripheral and lower lobe involvement.^[5]^ As COVID-19 mainly attacks people's respiratory system and causes lung damage, the rehabilitation of respiratory function is particularly important for patients. Additionally, critically ill patients even get hypoxic respiratory failure, dyspnea, and other symptoms. Taking into account the patient's increasing oxygen demand, respiratory support equipment such as ventilators is often used to maintain breathing. But mechanical ventilation places respiratory muscle like the diaphragm at a disadvantage causing weakness and other symptoms like breath stacking and lung injury.^[6]^

As research progresses, certain progress has been made in the virology, immunology, imaging, epidemiology, and clinical characteristics of the virus.^[1]^ Due to the temporary lack of specific antiviral therapeutics, adjuvant therapy has gradually become one of research directions on COVID-19. The prevention, treatment, and rehabilitation of COVID-19 in China have achieved great success with the assistance of traditional Chinese medicine (TCM).^[7]^ Acupuncture as a typical representative of TCM therapy is one of the most important methods to fight COVID-19 in China.^[8]^ Acupuncture is an effective, inexpensive, and safe treatment,^[9]^ with no side effects or infections have been reported^[10]^; it has been widely used in China for thousands of years and has been accepted worldwide. Contemporary acupuncture practice is commonly undertaken as part of the medical hospital system in modern China.^[11]^ Acupuncture were also used during the treatment of COVID-19 when the Shenzhen Traditional Chinese Medicine Team assisted Wuhan in Leishenshan Hospital.^[12]^ We can see asthmatic patients quickly felt smooth breathing and chest tightness relieved under the treatment of acupuncture. Acupuncture has been widely used in clinical practice to treat respiratory diseases,^[13]^ and has passed many randomized controlled trials (RCTs) tests.^[14]^ There are studies using needle moxibustion to treat chronic obstructive pulmonary disease patients with respiratory muscle fatigue, which has a significant improvement effect.^[15]^ In some studies, acupuncture combined with conventional western medicine treatment can improve the oxygenation index of mechanically ventilated patients, and the effect is better than pure western medicine treatment. Some study found that acupuncture can increase oxygen partial pressure, blood oxygen saturation, and clinical symptoms. The convalescence is significantly accelerated, and the time spent on ventilator can be significantly reduced.^[16]^ For patients who use ventilator, the incidence of complications, and intubation rate are also significantly reduced.

In summary, for COVID-19 patients after ventilator weaning Acupuncture can be used for adjuvant treatment in cases of decreased respiratory function and low oxygen saturation. It can also relax respiratory muscles to improve respiratory function. Acupuncture could also produce the local and systemic anti-inflammatory effect on COVID-19 through the activation of cholinergic anti-inflammatory pathway.^[17]^ For COVID-19, acupuncture can not only improve respiratory function, but also relieve anxiety and depression maximizing function retention and improving quality of life.^[18]^ Therefore, acupuncture has a certain rehabilitative effect on both respiratory disorders caused by COVID-19 and respiratory problems caused by mechanical ventilation. However, there has been no systematic review on the safety and effectiveness of acupuncture on respiratory rehabilitation for COVID-19 ventilator support patient after ventilator weaning. Therefore, in this meta-analysis review, our goal is to systematically review the safety and effectiveness of acupuncture on the recovery of respiratory after weaning from COVID-19 ventilator support patients, thereby improving the physiological function and quality of life.

## 2 Methods

Our study protocol has been registered in the PROSPERO and the registration number is CRD42020206889. The protocol follows the Cochrane Handbook for Systematic Reviews of Interventions and the Preferred Reporting Items for Systematic Reviews and Meta-Analysis Protocol statement guidelines. https://www.ncbi.nlm.nih.gov/pmc/articles/PMC7360276/ - R15

### 2.1 Inclusion criteria

#### 2.1.1 Study type

This review will include clinical RCTs. However, non-random or incorrectly randomized clinical research literature, reviews, experimental reports, clinical case reports, and animal research literature will be excluded.

#### 2.1.2 Participant types

Inclusion criteria:

(1) Patients who suffered from viral pneumonia caused by the COVID-19 and use a ventilator during treatment will be included, Regardless of the time of mechanical ventilation. Diagnosis of COVID-19 is based on the international or Chinese diagnostic criteria for COVID-19.^[19,20]^
(2) Do not consider the section whether in the intensive care unit, intermediate respiratory unit, general ward or rehabilitation facility will be involved in this meta-analysis.
(3) There is no restriction on the method of therapy, whether with standard oxygen therapy, High-flow Nasal Oxygen, Non-invasion positive pressure ventilation, invasive positive pressure ventilation or extra-corporeal membrane oxygenation.
(4) Also there will be no restrictions with respect to gender, age, education, economic status, or ethnicity.

Exclusion criteria: those with severe complications will be excluded.

#### 2.1.3 Types of interventions

Acupuncture will be performed in the treatment group, combined with other treatments, including respiratory rehabilitation training, routine therapy, and so on.^[21]^ Patients in the control group will receive other therapeutic approaches other than acupuncture, including respiratory rehabilitation training, routine therapy, placebo, and so on.

#### 2.1.4 Primary outcomes

The primary outcomes of interest will be maximum inspiratory pressure, maximum expiratory pressure, maximum inspiratory pressure/maximum expiratory pressure, arterial partial pressure of oxygen/fraction of inspired oxygen ratio, blood oxygen saturation, forced expiratory volume in 1 second, forced vital capacity, forced expiratory volume in 1 second/forced vital capacity, and Borg scale scores. The incidence rate of adverse events (defined as harmful reactions unrelated to the therapy) will also be assessed.

#### 2.1.5 Secondary outcomes

Additional outcomes of patient's condition are as follows: Changes of patient's condition:

1. Time until computed tomography image improved;
2. Time until cough reported as mild or absent;
3. Time until dyspnea reported as mild or absent;
4. Frequency of requiring respiratory

### 2.2 Search methods to identify studies

#### 2.2.1 Search strategy

The following electronic bibliographic databases will be searched to identify relevant studies: PubMed, EMBASE, MEDLINE, the Cochrane Library, Chinese National Knowledge Infrastructure, Chinese Biomedical Literature Database, Chinese Science and Technology Periodical Database, Wanfang Database, Clinical Trials and Chinese Clinical Trial Registry. The same terms in China will be conducted in Chinese databases. The searching strategy for PubMed is shown in Table 1.

**Table 1:** Preliminary search strategy in PubMed.

Search	Query
1	“Acupuncture”[Mesh]
2	(((((((((((((Pharmacopuncture[Title/Abstract])) OR (Acupuncture Therapy[Title/Abstract])) OR (Acupuncture Points[Title/Abstract])) OR (electroacupuncture[Title/Abstract])) OR (dermal needle[Title/Abstract])) OR (needle[Title/Abstract])) OR (acupoint[Title/Abstract])) OR (manual acupuncture[Title/Abstract])) OR (scale acupuncture[Title/Abstract])) OR (acutherapy[Title/Abstract])) OR (fire-needle[Title/Abstract])) OR (warm-needle[Title/Abstract])) OR (hydro-acupuncture[Title/Abstract])
3	#1 OR #2
4	(((((((((((COVID-19[Title/Abstract]) OR (2019 novel coronavirus disease[Title/Abstract])) OR (COVID19[Title/Abstract])) OR (COVID-19 pandemic[Title/Abstract])) OR (SARS-CoV-2 infection[Title/Abstract])) OR (COVID-19 virus disease[Title/Abstract])) OR (2019 novel coronavirus infection[Title/Abstract])) OR (2019-nCoV infection[Title/Abstract])) OR (coronavirus disease 2019[Title/Abstract])) OR (coronavirus disease-19[Title/Abstract])) OR (2019-nCoV disease[Title/Abstract])) OR (COVID-19 virus infection[Title/Abstract])
5	“Ventilator Weaning”[Mesh]
6	(((((Weaning, Ventilator[Title/Abstract]) OR (Respirator Weaning[Title/Abstract])) OR (Weaning, Respirator[Title/Abstract])) OR (Mechanical Ventilator Weaning[Title/Abstract])) OR (Ventilator Weaning, Mechanical[Title/Abstract])) OR (Weaning, Mechanical Ventilator[Title/Abstract])
7	#5 OR #6
8	“Respiratory Physiological Phenomena”[Mesh]
9	(((((((((((((((((Respiration[Title/Abstract]) OR (Breathing[Title/Abstract])) OR (Respiratory Function[Title/Abstract])) OR (Lung Injury[Title/Abstract])) OR (Injuries, Lung[Title/Abstract])) OR (Injury, Lung[Title/Abstract])) OR (Pulmonary Injury[Title/Abstract])) OR (Injuries, Pulmonary[Title/Abstract])) OR (Injury, Pulmonary[Title/Abstract])) OR (Pulmonary Injuries[Title/Abstract])) OR (Lung Injuries[Title/Abstract])) OR (Chronic Lung Injury[Title/Abstract])) OR (Chronic Lung Injuries[Title/Abstract])) OR (Lung Injuries, Chronic[Title/Abstract])) OR (Lung Injury, Chronic[Title/Abstract])) OR (Respiratory rehabilitation[Title/Abstract])) OR (pulmonary rehabilitation[Title/Abstract])) OR (pulmonary function[Title/Abstract])
10	#8 OR #9
11	“randomized controlled trial”[Publication Type] OR “randomized”[Title/Abstract] OR “placebo”[Title/Abstract]
12	#3 AND #4 AND #7 AND #10 AND #11

### 2.3 Data collection and analysis

#### 2.3.1 Selection of studies

**Figure 1:**
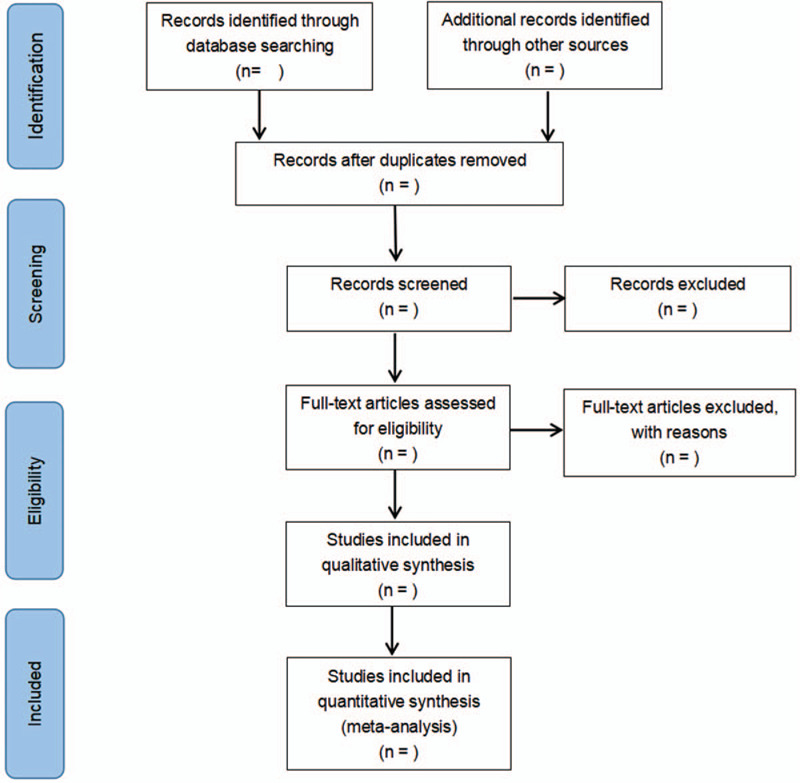
Figure is the PRISMA flow chart presenting details of the selection process. PRISMA = preferred reporting items for systematic review and meta-analysis.

We will use EndNote X9 software to manage the records of searched electronic databases. The full text of relevant studies will be reviewed for study inclusion, in accordance with the inclusion criteria. Two reviewers independently select studies, and any disagreement between the 2 reviewers should be consulted by a third reviewer to reach a consensus. We will remove repetitive articles at first and exclude irrelevant studies based on the title, abstract, and the full text. The study selection process is demonstrated in a Preferred Reporting Items for Systematic Reviews and Meta-Analysis flow diagram (Fig. 1).

#### 2.3.2 Data extraction and management

A standardized form will be used by 2 reviewers to extract data independently, and disagreements between them should be solved with the help of a third reviewer. The detailed extraction information are as follows: the first author, year of publication, study design, sample characteristics, number of participants, experimental and control interventions, intervention time, outcomes, results, and adverse reactions. We will try our best to contact the corresponding authors of the studies through email to deal with missing data. And the study will be further excluded without adequate information. If there is any dispute in the data extraction process, it will be submitted to a third reviewer for processing.

#### 2.3.3 Evaluation of bias risk in included studies

Two investigators will independently evaluate the bias risks among enrolled researches according to the Cochrane Collaboration. Discrepancy will be resolved by discussion and judgment by an arbiter. The following 7 aspects in all RCTs will be evaluated: outcome assessment blinding, participants and personnel blinding, selective outcome reporting, allocation concealment, inadequate outcome data, generation of random sequences as well as other potential sources of bias. The bias risk in each aspect will be assessed and divided into 3 levels: low risk, high risk, and unclear risk.

#### 2.3.4 Data synthesis and statistical analysis

Review Manager V.5.3 (Cochrane Collaboration) or Stata V.16.0 software will be used to conduct this meta-analysis. The mean difference or standardized mean difference with 95% confidence intervals is used to calculate continuous variables. Dichotomous data will be reported as risk ratios with their 95% confidence intervals. *P* < .05 will be considered to be statistically significant.

#### 2.3.5 Heterogeneity evaluation

Heterogeneity will be assessed by the *χ*^2^ test and the *I*^2^ test. If *P* > .10 and *I*^2^ < 50%, the heterogeneity is acceptable and a fixed-effect model will be used for data analysis. If *P* < .10 and *I*^2^ ≥ 50%, we will search for the reasons for the high heterogeneity and use a random-effects model for data analysis.

#### 2.3.6 Publication bias

The publication bias will be evaluated by funnel plots by determining whether there are 10 or more studies with the same outcome. In the case of asymmetric funnel plot, subgroup analysis or sensitivity analysis will be performed to investigate possible causes.

#### 2.3.7 Subgroup analysis

If there is a large heterogeneity in the included studies, subgroup analyses will be performed on different types of acupoint and different treatment locations, such as, intensive care unit, intermediate respiratory unit, general ward, or rehabilitation facility.

#### 2.3.8 Sensitivity analysis

Sensitivity analysis is conducted by excluding studies one by one, so that we can determine the source of heterogeneity.

#### 2.3.9 Assessment of evidence quality

The quality of evidence will be assessed based on the Grading of Recommendations Assessment, Development, and Evaluation system. The evidence will be adjusted to 4 levels: high, moderate, low, or very low.

#### 2.3.10 Ethics and dissemination

The data used in this systematic review will be collected from published studies. Based on this, the study does not require ethical approval.

## 3 Discussion

COVID-19 is one of the greatest challenges facing people around the world in the 21st century, with respiratory symptoms most pronounced. Patients with symptoms such as severe respiratory failure are often supported by mechanical ventilation. The damage of COVID-19 to the respiratory system and the use of a ventilator may cause adverse consequences such as airway damage, atelectasis, respiratory muscle paralysis, and a decrease in lung function. The combination of this 2 cause further damage to respiratory comparing with pure COVID-19. So the respiratory rehabilitation of COVID-19 patients who get ventilator support during the treatment is particularly important. At present, there are many studies on the diagnosis, prevention, and treatment of COVID-19 in TCM, especially on the pathogenesis, clinical syndromes, and treatment plan of respiratory diseases. Acupuncture which is a kind of well-known traditional Chinese external treatment, has been used as adjuvant treatment for it in China.^[22]^ Acupuncture can improve the symptoms of respiratory tract obstruction, the blood metabolism of lung and bronchial tissue, decrease efficiently the recovery time of respiratory muscle, and so on. Acupuncture can also improve immunity and relieve anxiety with specific acupoints, which can enhance the quality of life of such patients. The cognitive status, musculoskeletal system, and respiratory system of the patients after mechanical ventilation are poor, and their physical strength is not good. Therefore, the previous postural changes and breathing exercises are not always applicable. Acupuncture, by contrast, can be done in a fixed position, which can reduce the physical exertion of weak patients and play the above effects. Such treatments can be effective and relieve the pressure on medical funding at the same time. The results of this systematic review and meta-analysis may establish a better approach to adjuvant treatment for COVID-19 and analyze application of acupuncture therapy on COVID-19 patients with ventilator support patient after ventilator weaning.

## 4 Conclusion

The conclusion of this study will give evidence to prove the safety and effectiveness of acupuncture for rehabilitation on respiratory after weaning from the ventilator during the treatment of COVID-19.

## Author contributions

**Conceptualization:** Wenxin Chi, Ziyu Luo, Xiangyu Zhu.

**Data curation:** Ying Chen, Lina Wang, Yu Zhang.

**Formal analysis:** Xiangyu Zhu, Ying Chen, Lina Wang.

**Methodology:** Wenxin Chi, Ying Chen, Lina Wang, Ziyu Luo.

**Project administration:** Wenxin Chi.

**Validation:** Lina Wang.

**Writing – original draft:** Wenxin Chi.

**Writing – review and editing:** Wenxin Chi, Ziyu Luo, Xiangyu Zhu.

## References

[1] Tan AS Nerurkar SN Tan WCC The virological, immunological, and imaging approaches for COVID-19 diagnosis and research. SLAS Technol 2020;25:522–44. 32808850 10.1177/2472630320950248 PMC7435207

[2] Wang L Wang Y Ye D Review of the 2019 novel coronavirus (SARS-CoV-2) based on current evidence [published correction appears in Int J Antimicrob Agents. 2020 Sep;56 (3):106137]. Int J Antimicrob Agents 2020;55:105948. 32201353 10.1016/j.ijantimicag.2020.105948 PMC7156162

[3] Gulati A Pomeranz C Qamar Z A comprehensive review of manifestations of novel coronaviruses in the context of deadly COVID-19 global pandemic. Am J Med Sci 2020;360:5–34. 32620220 10.1016/j.amjms.2020.05.006 PMC7212949

[4] McFadyen JD Stevens H Peter K The emerging threat of (micro)thrombosis in COVID-19 and its therapeutic implications. Circ Res 2020;127:571–87. 32586214 10.1161/CIRCRESAHA.120.317447 PMC7386875

[5] Shi H Han X Jiang N Radiological findings from 81 patients with COVID-19 pneumonia in Wuhan, China: a descriptive study. Lancet Infect Dis 2020;20:425–34. 32105637 10.1016/S1473-3099(20)30086-4 PMC7159053

[6] Hamahata NT Sato R Daoud EG Go with the flow-clinical importance of flow curves during mechanical ventilation: a narrative review. Can J Respir Ther 2020;56:11–20. 32844110 10.29390/cjrt-2020-002 PMC7427988

[7] Xu X Shi YN Wang RY Home-based traditional Chinese medicine nursing interventions for discharged patients with COVID-19: a rapid review of Chinese guidelines. Integr Med Res 2020;9:100479. 32766114 10.1016/j.imr.2020.100479 PMC7365124

[8] Liu B Wang H Zhou ZY Analysis on the theory and clinical ideas of acupuncture and moxibustion for the prevention and treatment of coronavirus disease 2019. Zhongguo Zhen Jiu 2020;40:571–5. 32538003 10.13703/j.0255-2930.20200305-k0004

[9] Arthur YF David WM Bonnie B Acupuncture's role in solving the opioid epidemic: evidence, cost-effectiveness, and care availability for acupuncture as a primary, non-pharmacologic method for pain relief and management—white paper 2017. J Integrative Med 2017;6:411–25. 10.1016/S2095-4964(17)60378-9 29103410

[10] Chien TJ Liu CY Fang CJ The effect of acupuncture in breast cancer-related lymphoedema (BCRL): a systematic review and meta-analysis. Integr Cancer Ther 2019;18:1534735419866910 Erratum in: Integr Cancer Ther. 2019 Jan–Dec;18:1534735419875326. 31387468 10.1177/1534735419866910 PMC6686319

[11] Zhang Q Xu X Sun S Efficacy of acupuncture and moxibustion in adjuvant treatment of patients with novel coronavirus disease 2019 (COVID-19): a protocol for systematic review and meta analysis. Medicine (Baltimore) 2020;99:e21039. 32664113 10.1097/MD.0000000000021039 PMC7360278

[12] Gong YB Yang ZL Liu Y Two cases of coronavirus disease 2019 (COVID-19) treated with the combination of acupuncture and medication in bedridden patients. World J Acupunct Moxibustion 2020;30:171–4. 32837108 10.1016/j.wjam.2020.07.005 PMC7367001

[13] Li CX Li ZX Wang D Advances in the clinical research on acupuncture in treatment of respiratory diseases. Zhen Ci Yan Jiu 2020;45:169–72. 32144930 10.13702/j.1000-0607.1804086

[14] Li Y Xiong C Zeng Y Acupuncture treatment of lung-spleen Qi deficiency in stable chronic obstructive pulmonary disease: a randomized, open-label, controlled trial. J Tradit Chin Med 2019;39:885–91. 32186160

[15] Fernández-Jané C Vilaró J Fei Y Acupuncture techniques for COPD: a systematic review. BMC Complement Med Ther 2020;20:138. 32375775 10.1186/s12906-020-02899-3 PMC7323612

[16] Chang L Xian W Jingyu Z Discussion on the advantages of acupuncture and moxibustion in the prevention and treatment of COVID-19. J Hainan Med Univ 2020;26:1527–30. +1536.

[17] He W Shi XS Zhang ZY Discussion on the effect pathways of preventing and treating coronavirus disease 2019. Zhong guo Zhen Jiu 2020;40:799–802. 10.13703/j.0255-2930.20200305-0001 32869585

[18] Jia H Han Z Zhang K Acupuncture and related interventions for anxiety in coronavirus disease 2019: a protocol for systematic review and meta-analysis. Medicine (Baltimore) 2020;99:e21317. 32791723 10.1097/MD.0000000000021317 PMC7387029

[19] National Health Commission of the People's Republic of China.<Guideline on diagnosis and treatment of COVID-19 (Trial 6th edition). Available at: http://www.nhc.gov.cn/xcs/zhengcwj/202002/8334a8326dd94d329df351d7da8aefc2.shtml. [access date February 23, 2020].

[20] Ahn DG Shin HJ Kim MH Current status of epidemiology, diagnosis, therapeutics, and vaccines for novel coronavirus disease 2019 (COVID-19). J Microbiol Biotechnol 2020;30:313–24. 32238757 10.4014/jmb.2003.03011 PMC9728410

[21] Jin YH Cai L Cheng ZS A rapid advice guideline for the diagnosis and treatment of 2019 novel coronavirus nCoV) infected pneumonia (standard version). Mil Med Res 2020;7:4. 32029004 10.1186/s40779-020-0233-6 PMC7003341

[22] Zhang HL Huo ZJ Wang HN Acupuncture ameliorates negative emotion in PCOS patients: a randomized controlled trial. Zhongguo Zhen Jiu 2020;40:385–90. 32275367 10.13703/j.0255-2930.20191231-k0005

